# Response to treatment in psoriatic arthritis, the effect of age: analysis of patients receiving ustekinumab in the PsABio real-world study

**DOI:** 10.1186/s13075-023-03078-8

**Published:** 2023-06-09

**Authors:** Laure Gossec, Elke Theander, Soumya D. Chakravarty, Paul Bergmans, Frederic Lavie, Wim Noël, Mohamed Sharaf, Stefan Siebert, Josef S. Smolen

**Affiliations:** 1 Faculty of Medicine, Sorbonne Université, INSERM, IPLESP, Paris, France; 2 Department of Rheumatology, Pitié-Salpêtrière Hospital, AP-HP, 47-83 Bd Hôpital, 75013 Paris, France; 3 Department of Medical Affairs, Janssen-Cilag AB, Solna, Sweden; 4 Department of Immunology, Janssen Scientific Affairs, LLC, Horsham, PA USA; 5 Department of Rheumatology, Drexel University College of Medicine, Philadelphia, PA USA; 6 Department of Biostatistics, Janssen-Cilag BV, Breda, Netherlands; 7 Department of Medical Affairs, Janssen-Cilag, Cedex, France; 8 Department of Medical Affairs, Janssen Pharmaceuticals NV, Beerse, Belgium; 9 Department of Medical Affairs, Janssen, Dubai, United Arab Emirates; 10 School of Infection & Immunity, College of Medical, Veterinary & Life Sciences, University of Glasgow, Glasgow, UK; 11 Department of Internal Medicine III, Medical University of Vienna, Vienna, Austria

**Keywords:** Interleukins, Psoriatic arthritis, Disease modifying anti-rheumatic drugs

## Abstract

**Background:**

This post-hoc analysis of PsABio (NCT02627768) evaluated safety, effectiveness and treatment persistence in patients < 60 and ≥ 60 years of age receiving ustekinumab over 3 years.

**Methods:**

Measures included adverse events (AE), clinical Disease Activity Index for Psoriatic Arthritis (cDAPSA) low disease activity (LDA) including remission, Psoriatic Arthritis Impact of Disease-12 (PsAID-12), Minimal Disease Activity, dactylitis, nail/skin involvement and time to treatment stop. Data were analysed descriptively.

**Results:**

Overall, 336 patients < 60 and 103 ≥ 60 years received ustekinumab, with a similar gender balance. A numerically lower proportion of younger patients reported at least one AE: 124/379 (32.7%) vs 47/115 (40.9%) for patients < 60 and ≥ 60 years, respectively. Serious AEs were low (< 10%) in both groups. At 6 months, the proportion of patients with cDAPSA LDA was 138/267 (51.7%) and 35/80 (43.8%) for patients < 60 and ≥ 60 years, respectively, with the effectiveness being maintained through 36 months. PsAID-12 mean scores reduced for both groups from a baseline mean of 5.73 and 5.61 for patients < 60 and ≥ 60 years, respectively, to 3.81 and 3.88, respectively, at 6 months, and 2.02 and 3.24, respectively, at 36 months. Regarding treatment persistence, 173/336 (51.5%) vs 47/103 (45.6%) patients < 60 and ≥ 60 years, respectively, stopped or switched treatment.

**Conclusion:**

Fewer AEs were observed over 3 years for younger versus older patients with PsA. There were no clinically meaningful treatment response differences. Persistence was numerically higher in the older age group.

**Supplementary Information:**

The online version contains supplementary material available at 10.1186/s13075-023-03078-8.

## Background

Psoriatic arthritis (PsA) affects patients usually from the age of 30 onwards [1]. Older patients have often accumulated multiple comorbidities, including cardiovascular (CV) disease, therefore the safety and tolerability of effective biologic disease-modifying antirheumatic drugs (bDMARDs) for the treatment of PsA are particularly important in older patients.

The long-term safety, effectiveness and treatment persistence of bDMARDs for PsA has not been thoroughly explored, especially in older patients. Age ≥ 65 years is often considered the cut-off for people termed ‘elderly’; a cut-off for ‘older’ patients of ≥ 60 years of age has also been used in rheumatoid arthritis studies in the past [2, 3].

Ustekinumab is a human monoclonal antibody that targets the shared p40 subunit of interleukin (IL)-12 and IL-23, interfering with intracellular signalling and cytokine secretion to inhibit immune cell activation. It has been shown to have a good safety profile and be efficacious in the management of psoriasis [4, 5] and PsA [6, 7] in a number of company-sponsored Phase 2 and 3 trials. In the PsABio study, [8–10] ustekinumab has shown similar effectiveness as tumour necrosis factor inhibitors (TNFi) over a three-year period.

In this post-hoc analysis, we analysed the safety, effectiveness and persistence of ustekinumab for patients < 60 and ≥ 60 years of age in the PsABio study over 3 years.

## Methods

### Study design and patient population

PsABio (NCT02627768) was a multinational, prospective, real-world, observational study of patients with PsA who started either ustekinumab (an IL-12/IL-23 p40 inhibitor) or a new TNFi as a first-, second- or third-line biologic treatment. This post-hoc analysis focuses on ustekinumab treatment only. The PsABio study has been described elsewhere [8–10]. Study duration per participant was up to 36 months or until treatment stop/switch, with follow-up every 6 months (± 3 months to align with standard clinical practice).

### Assessments

#### Safety

All adverse events (AEs) that started at or after ustekinumab treatment, up to 91 days (the dispension interval) after the last dose, are reported. Malignancy AEs were recorded with a 1-year lag time after first exposure to ustekinumab and until study end for all patients, independent of ustekinumab stop date.

AEs of special interest were serious infections and opportunistic infections (with possible relatedness), progressive multifocal leukoencephalopathy, posterior reversible encephalopathy syndrome, anaphylactic/anaphylactoid reactions, pustular psoriasis, exfoliative dermatitis, erythrodermic psoriasis, CV events (myocardial infarction, stroke, CV death and death of unknown cause, and any other CV event), severe depression including suicidality and malignancies.

#### Effectiveness

Effectiveness measures included clinical Disease Activity Index for Psoriatic Arthritis (cDAPSA) low disease activity (LDA) including remission, herein described as cDAPSA LDA; Minimal Disease Activity (MDA) including Very Low Disease Activity (VLDA), herein described as MDA; Psoriatic Arthritis Impact of Disease-12 (PsAID-12), including total PsAID, and total PsAID < 4 for patients with baseline ≥ 4; dactylitis, enthesitis, nail lesion and skin lesion resolution (body surface area rate (BSA): clear/almost clear skin, < 3% but not clear/almost clear; 3–10%; and > 10%).

Components of the American College of Rheumatology (ACR) response criteria were assessed including tender joint count, 68 joints (TJC68), swollen joint count, 66 joints (SJC66), Health Assessment Questionnaire-Disability Index (HAQ-DI), C-reactive protein (CRP), Physician’s Global Assessment of Disease Activity (PGA-PsA), Patient’s Global Assessment of Disease Activity-Visual Analogue Scale (PtGA-VAS) and patient assessment of pain-VAS (Pain-VAS).

#### Treatment persistence

Treatment duration was calculated as first to last treatment dose plus one dispensing interval if no other bDMARD was started, or to withdrawal/stop/switch of treatment, death or patient lost to follow-up, whichever occurred first.

### Statistical analysis

This analysis was exploratory; no predefined hypotheses were tested and no adjustment for multiplicity was applied. Data were analysed by descriptive statistics only including 95% confidence interval (CI). The CIs are provided in Tables and Figures unless reported in the text. In addition to observed case analysis, a last observation carried forward (LOCF) endpoint was also created. Kaplan-Meier plots with log rank tests to compare age groups were calculated for time to ustekinumab treatment stop/switch.

To avoid potential selection bias, all eligible patients were to be offered enrolment for data collection in the study.

To try to understand treatment persistence in ustekinumab- and TNFi-treated patients, for both treatments, it was planned to create several subgroups. If subgroups were created of 100 patients, the width of the 95% CI of the observed proportion would vary from 0.19 for a sample proportion of 0.50 to 0.12 for a sample proportion of 0.90 (PASS 11.0.9; CI for 1 proportion). The widths of these 95% CIs were considered relevant from clinical perspective.

## Results

### Study population

The baseline set consisted of 458 patients: 353 < 60 years and 105 ≥ 60 years; the effectiveness set of 439 patients: 336 < 60 years and 103 ≥ 60 years; and the safety set of 494 patients: 379 < 60 years and 115 ≥ 60 years. The safety set included all eligible patients who used ustekinumab, either as initial treatment or after switching from TNFi.

### Baseline demographic and disease characteristics (effectiveness data set)

At baseline, there was a similar proportion of male subjects in both age groups: 43.8% < 60 years vs 43.7% ≥ 60 years (Table 1). As expected, disease duration was shorter in patients < 60 years vs ≥ 60 years (6.88 vs 9.51 years), and there was a lower proportion (30.7 vs 78.6%, respectively) of CV/metabolic syndrome comorbidities (hypertension, myocardial infarction, congestive heart failure, stroke or transient ischemic attack, peripheral vascular disease, hyperlipidaemia, type 1 or 2 diabetes or angina pectoris). Comparable scores were reported for mean cDAPSA, mean total PsAID-12 and for components of the ACR response criteria, except mean CRP, which was lower in patients < 60 years vs ≥ 60 years. A similar proportion of patients < 60 years vs ≥ 60 years had nail and skin involvement. A higher proportion of patients < 60 years vs ≥ 60 years had dactylitis and enthesitis (Table 1).

**Table 1:** Patient demographic and disease characteristics at baseline – effectiveness set

	Total patients *N* = 439	Age < 60 years *N* = 336	Age ≥ 60 years *N* = 103
Age, years, mean (95% CI) Median [range]	51.1 (49.9; 52.2) 51.0 [18; 86]	46.1 (45.1; 47.1) 48.0 [18; 59]	67.3 (66.0; 68.5) 66.0 [60; 86]
Male subjects, n (%)	192 (43.7)	147 (43.8)	45 (43.7)
Time since initial diagnosis, years, mean (95% CI)	7.67 (6.53; 8.81)	6.88 (6.07; 7.69)	9.51 (7.59; 11.43)
Methotrexate / corticosteroids at baseline, n (%)	132 (30.1) / 88 (20.0)	102 (30.4) / 66 (19.6)	30 (29.1) / 22 (21.4)
CV/metabolic syndrome comorbidities, n (%)	184 (41.9)	103 (30.7)	81 (78.6)
cDAPSA score, mean (95% CI) low^a^/ moderate / high, n (%)	30.42 (28.37; 32.47) 48 (13.0) / 145 (39.2) / 177 (47.8)	30.50 (28.09; 32.91) 39 (13.8) / 112 (39.6) / 132 (46.6)	30.17 (26.26; 34.08) 9 (10.3) / 33 (37.9) / 45 (51.7)
MDA, n (%) (95% CI)	17 (4.7) (2.8; 7.4)	14 (5.0) (2.8; 8.3)	3 (3.6) (0.7; 10.1)
Total PsAID-12 score, mean (95% CI)	5.70 (5.49; 5.91)	5.73 (5.48; 5.98)	5.61 (5.19; 6.03)
Dactylitis, n (%) (95% CI)	74 (18.1) (14.5; 22.2)	63 (20.0) (15.7; 24.8)	11 (11.8) (6.1; 20.2)
Enthesitis, n (%) (95% CI)	194 (47.8) (42.8; 52.8)	157 (50.3) (44.6; 56.0)	37 (39.4) (29.4; 50.0)
Nail lesions, n (%) (95% CI)	180 (45.9) (40.9; 51.0)	142 (46.7) (41.0; 52.5)	38 (43.2) (32.7; 54.2)
Body surface area rate, n (%) (95% CI)			
Clear/almost clear skin	106 (29.4) (24.7; 34.4)	84 (29.8) (24.5; 35.5)	22 (27.8) (18.3; 39.1)
< 3% but not clear/almost clear	36 (10.0) (7.1; 13.5)	30 (10.6) (7.3; 14.8)	6 (7.6) (2.8; 15.8)
3–10%	124 (34.3) (29.5; 39.5)	94 (33.3) (27.9; 39.2)	30 (38.0) (27.3; 49.6)
> 10%	95 (26.3) (21.8; 31.2)	74 (26.2) (21.2; 31.8)	21 (26.6) (17.3; 37.7)
TJC68, mean (95% CI)	12.3 (11.1; 13.6)	12.5 (11.0; 13.9)	11.7 (9.3; 14.1)
SJC66, mean (95% CI)	5.8 (5.0; 6.6)	5.8 (4.9; 6.8)	5.9 (4.2; 7.5)
HAQ-DI score, mean (95% CI)	1.1 (1.1; 1.2)	1.1 (1.0; 1.2)	1.2 (1.1; 1.4)
CRP, mg/dL, mean (95% CI)	1.3 (1.0; 1.7)	1.1 (0.8; 1.3)	2.2 (1.0; 3.4)
PGA-PsA, mean (mm) (95% CI)	53.8 (51.8; 55.7)	53.8 (51.6; 56.0)	53.4 (48.9; 58.0)
PtGA-VAS over past week, mean (mm) (95% CI)	60.7 (58.4; 63.1)	59.9 (57.2; 62.6)	63.3 (58.2; 68.4)
Pain-VAS over past week, mean (mm) (95% CI)	60.4 (58.0; 62.8)	59.5 (56.7; 62.3)	63.2 (58.3; 68.1)

*cDAPSA* clinical Disease Activity Index for Psoriatic Arthritis, *CI* confidence interval, *CRP* C-reactive protein, *CV* cardiovascular, *HAQ-DI* Health Assessment Questionnaire-Disability Index, *MDA* Minimal Disease Activity, *Pain-VAS* Patient assessment of pain-VAS, *PGA-PsA* Physician Global Assessment of Disease Activity-Psoriatic Arthritis, *PsAID-12* 12-item Psoriatic Arthritis Impact of Disease questionnaire, *PtGA* Patient’s Global Assessment of Disease Activity, *SJC66* Swollen joint count, 66 joints, *TJC68* Tender joint count, 68 joints, *VAS* Visual Analog Scale

^a^includes remission

### Safety

There were 991.3 patient years of ustekinumab exposure in total: 745.1 years for patients < 60 years and 246.2 years for patients ≥ 60 years (Table 2). In patients who used ustekinumab as initial treatment or after switching from TNFi, a numerically lower proportion reported at least one AE in the < 60 years group, 124/379 (32.7%) vs patients ≥ 60 years, 47/115 (40.9%). Likewise, serious AEs (SAEs) were reported by a numerically lower proportion of patients < 60 years, 20/379 (5.3%), vs patients ≥ 60 years, 11/115 (9.6%). AEs of special interest were recorded by 6/379 (1.6%) patients < 60 years and 7/115 (6.1%) patients ≥ 60 years, the most common of which were serious infections and opportunistic infections (with possible relatedness) in patients < 60 years (5/379 [1.3%]) and CV events in patients ≥ 60 years (5/115 [4.3%]). Malignancies were reported by 1/379 (0.3%) patients < 60 years and 5/115 (4.3%) ≥ 60 years (Table 2). The incidence rates of AEs of special interest per 100 patient-years were low in both groups (1.1 and 2.8, respectively) (Supplementary Fig. 1).

**Table 2:** Adverse events in patients with PsA according to age subgroup

Patients with ≥ 1 AE	Total *N* = 494	Age < 60 years *N* = 379	Age ≥ 60 years *N* = 115
Total patient years of exposure	991.3	745.1	246.2
Any AE^a^ n (%) (95% CI)	171 (34.6) (30.4; 39.0)	124 (32.7) (28.0; 37.7)	47 (40.9) (31.8; 50.4)
Any study treatment-related AE n (%) (95% CI)	84 (17.0) (13.78; 20.6)	69 (18.2) (14.5; 22.5)	15 (13.0) (7.5; 20.6)
Any SAE n (%) (95% CI)	31 (6.3) (4.3; 8.8)	20 (5.3) (3.3; 8.0)	11 (9.6) (4.9; 6.5)
Any study treatment-related SAE n (%) (95% CI)	7 (1.4) (0.6; 2.9)	6 (1.6) (0.6; 3.4)	1 (0.9) (0.0;4.7)
Any AE leading to withdrawal n (%) (95% CI)	43 (8.7) (6.4; 11.5)	37 (9.8) (7.0; 13.2)	6 (5.2) (1.9; 0.9)
Any AE of special interest n (%) (95% CI)	13 (2.6) (1.4; 4.5)	6 (1.6) (0.6; 3.4)	7 (6.1) (2.5; 12.1)
Serious infections and opportunistic infections (with possible relatedness)	6 (1.2) (0.4; 2.6)	5 (1.3) (0.4; 3.1)	1 (0.9) (0.0; 4.7)
CV events	6 (1.2) (0.4; 2.6)	1 (0.3) (0.0; 1.5)	5 (4.3) (1.4; 9.9)
Severe depression including suicidality	1 (0.2) (0.0; 1.0)	1 (0.3) (0.0; 1.5)	0
Malignancies	1 (0.2) (0.0; 1.0)	0	1 (0.9) (0.0; 4.7)
Any neoplasm AE (no lag time) n (%) (95% CI)	10 (2.0) (1.0; 3.7)	4 (1.1) (0.3; 2.7)	6 (5.2) (1.9; 11.0)
Benign neoplasm	4 (0.8) (0.2; 2.1)	3 (0.8) (0.2; 2.3)	1 (0.9) (0.0; 4.7)
Non-melanoma skin cancer	1 (0.2) (0.0; 1.1)	0	1 (0.9) (0.0; 4.7)
Malignancy (excl. non-melanoma skin cancer)	5 (1.0) (0.3; 2.3)	1 (0.3) (0.0; 1.5)	4 (3.5) (1.0; 8.7)
Any neoplasm AE (12-month lag time) n (%) (95% CI)	6 (1.2) (0.4; 2.6)	2 (0.5) (0.1; 1.9)	4 (3.5) (1.0; 8.7)
Benign neoplasm	3 (0.6) (0.1; 1.8)	2 (0.5) (0.1; 1.9)	1 (0.9) (0.0; 4.7)
Malignancy (excl. non-melanoma skin cancer)	3 (0.6) (0.1; 1.8)	0	3 (2.6) (0.5; 7.4)
Death	1 (0.2) (0.0; 1.1)	0	1 (0.9) (0.0; 4.7)

*AE* adverse event, *CI* confidence interval, *CV* cardiovascular, *PsA* psoriatic arthritis, *SAE* serious adverse event

^a^AEs do not include neoplasms unless stated

One 65-year-old male patient experienced a SAE leading to death (sudden death due to a CV event; this patient had pre-existing cardiac comorbidities including hypertension and stroke/transient ischemic attack and a BMI > 30 kg/m^2^).

### Effectiveness

#### cDAPSA

**Fig. 1:**
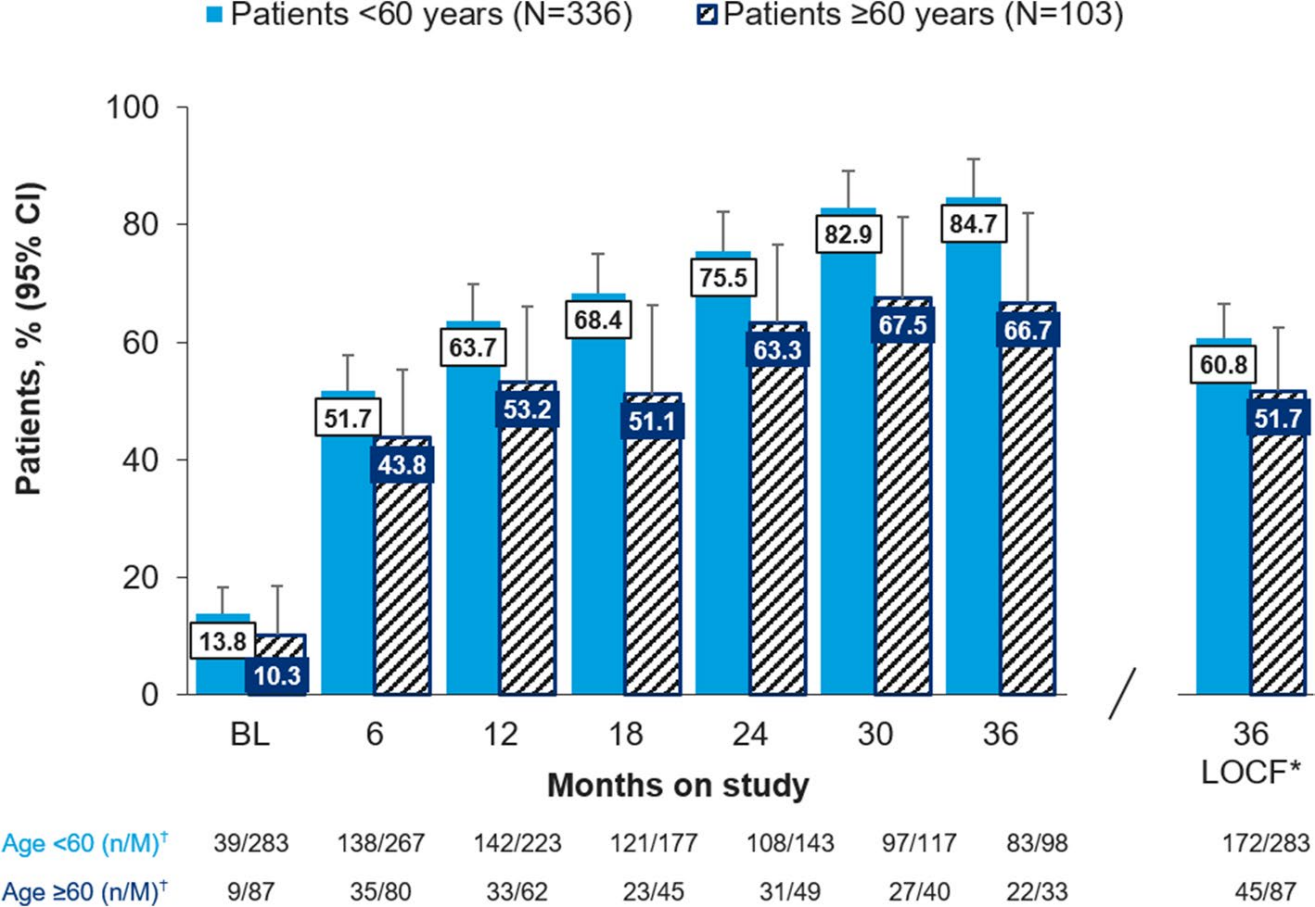
Proportion of patients achieving cDAPSA LDA including remission over time and by age subgroup, % (95% CI). Legend: BL, baseline; cDAPSA, clinical Disease Activity Index for Psoriatic Arthritis; CI, confidence interval; LDA, low disease activity; LOCF, last observation carried forward. *last observation carried forward, all other bars show observed case analysis; ^†^n, number of patients with cDAPSA low disease activity (including remission); M, number of patients with an assessment at that specific time point

Following ustekinumab treatment, at 6 months, the proportion of patients achieving cDAPSA LDA was 138/267 (51.7%) patients < 60 years and 35/80 (43.8%) patients ≥ 60 years from a baseline of 39/283 (13.8%) and 9/87 (10.3%) for patients < 60 years and ≥ 60 years, respectively. The proportion of patients increased at each 6-month time point for patients < 60 years, although this increase was not as pronounced for patients ≥ 60 years (Fig. 1).

#### MDA

**Fig. 2:**
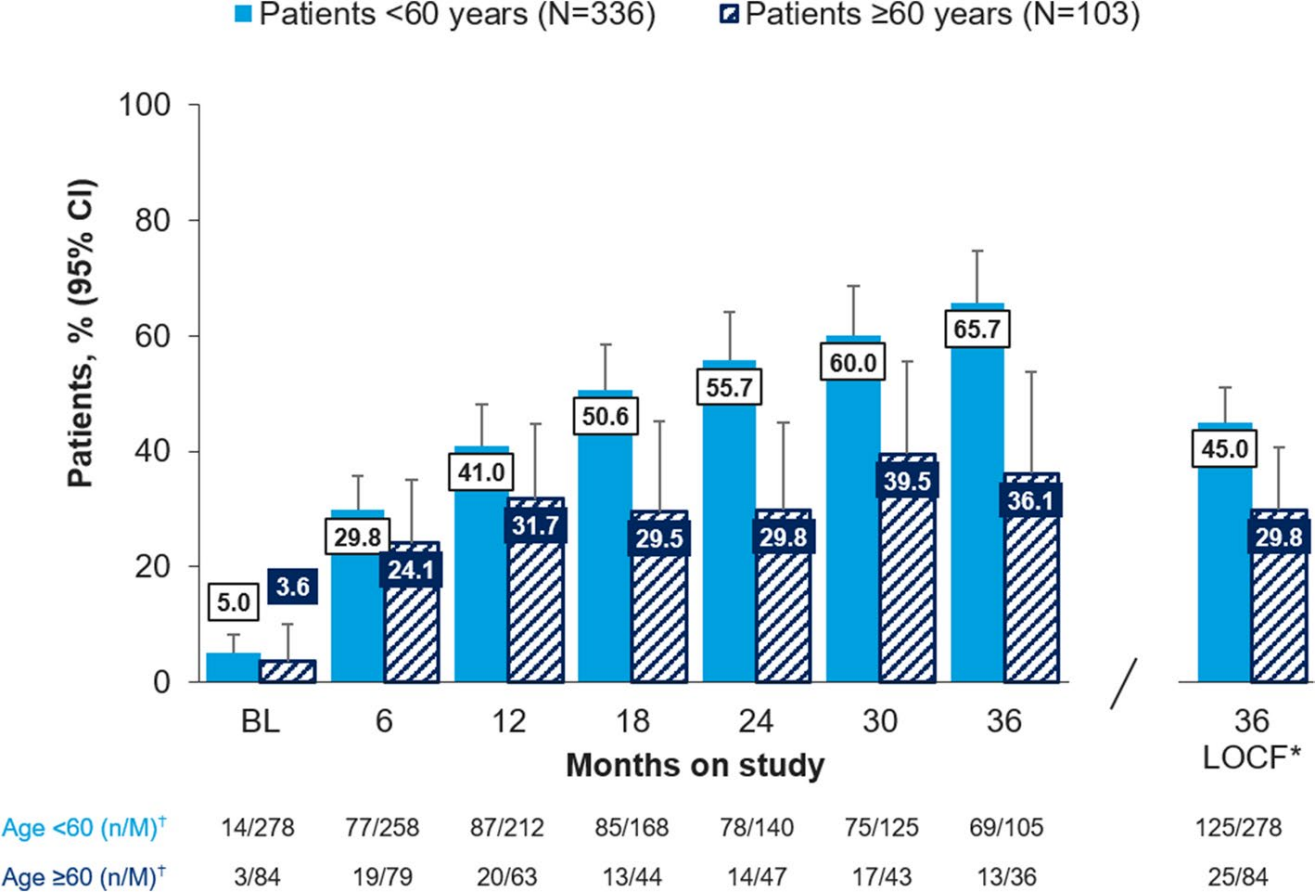
Proportion of patients achieving MDA including VLDA over time and by age subgroup, % (95% CI). Legend: BL, baseline; CI, confidence interval; LOCF, last observation carried forward; MDA, minimal disease activity; VLDA, very low disease activity. *last observation carried forward, all other bars show observed case analysis; ^†^n, number of patients with MDA including VLDA; M, number of patients with an assessment at that specific time point

The proportion of patients achieving MDA following ustekinumab treatment at 6 months was 77/258 (29.8%) patients < 60 years and 19/79 (24.1%) patients ≥ 60 years from a baseline of 14/278 (5.0%) and 3/84 (3.6%) for patients < 60 and ≥ 60 years, respectively. The proportion of patients in MDA increased at each 6-month time point for patients < 60 years. This 6-monthly increase was not observed in patients ≥ 60 years, however the proportion achieving the outcome was maintained to the end of the study (Fig. 2).

#### PsAID-12

**Fig. 3:**
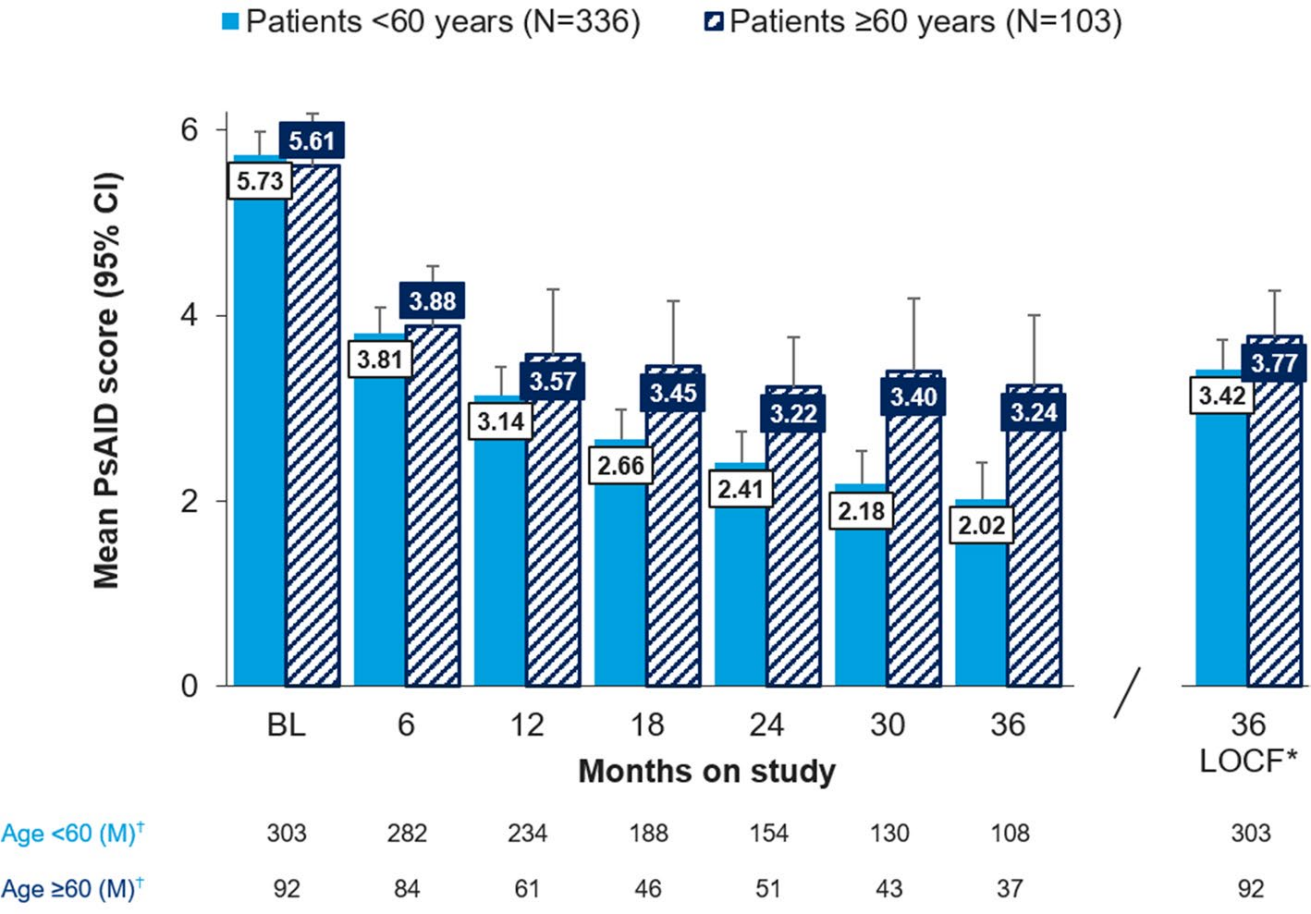
Total PsAID score over time and by age subgroup, Mean (95% CI). Legend: BL, baseline; CI, confidence interval; LOCF, last observation carried forward; PsAID, Psoriatic Arthritis Impact of Disease. *last observation carried forward, all other bars show observed case analysis; ^†^M, number of patients with an assessment at that specific time point

Impact of the disease, measured by mean total PsAID-12 scores, reduced for both groups from a baseline of 5.73 and 5.61 for patients < 60 and ≥ 60 years, respectively, to 3.81 and 3.88 at 6 months (< 4 is described as acceptable for patients [11]). While patients < 60 years continued improving at each 6-month time point, scores for patients ≥ 60 years appeared to plateau 18–36 months. At month 36, mean scores were 2.02 and 3.24 for patients < 60 years and ≥ 60 years, respectively (non-overlapping 95% CIs) (Fig. 3). For patients with PsAID ≥ 4 at baseline, 56/75 (74.6%) and 15/27 (55.6%) of patients < 60 years and ≥ 60 years, respectively, had a mean score < 4 at 36 months.

#### Dactylitis

At baseline, 63/315 (20.0%) patients < 60 years and 11/93 (11.8%) patients ≥ 60 years had evidence of dactylitis. Of these patients, 39/60 (65.0%) < 60 years and 10/11 (90.9%) ≥ 60 years achieved resolution of dactylitis at 6 months, increasing over time. By 18 months all remaining patients ≥ 60 years with dactylitis at baseline (8/8) had achieved resolution (maintained to 36 months), and 28/29 (96.6%) of patients < 60 years by 36 months (Supplementary Fig. 2).

#### Nail involvement

At baseline, 142/304 (46.7%) patients < 60 years and 38/88 (43.2%) patients ≥ 60 years had nail involvement. Of these patients, 46/135 (34.1%) < 60 years and 16/37 (43.2%) ≥ 60 years achieved resolution of nail lesions at 6 months. At 36 months, proportions of patients achieving resolution increased to 39/73 (53.4%) patients < 60 years and remained stable at 7/16 (43.8%) in patients ≥ 60 years (Supplementary Fig. 3).

#### Skin involvement

At baseline, the proportion of patients with BSA > 10% was 74/282 (26.2%; 95% CI 21.2, 31.8) and 21/79 (26.6%; 95% CI 17.3, 37.7) for patients < 60 years and ≥ 60 years, respectively. The proportion of patients with BSA > 10% decreased to 9/254 (3.5%; 95% CI 1.6, 6.6) and 3/73 (4.1%; 95% CI 0.9, 11.5) at 6 months and was maintained to 36 months, for patients < 60 years and ≥ 60 years, respectively. Accordingly, the proportion of patients with clear/almost clear skin increased at 6 months and the response was maintained over time in the < 60 years group (79/104 [76.0%; 95% CI 66.6, 83.8] at 36 months) and in the ≥ 60 years group (20/31 [64.5%; 95% CI 45.4, 80.8] at 36 months) (Supplementary Fig. 4).

#### ACR components

Over the course of the study, scores generally decreased (i.e. improved) for the individual ACR components (Supplementary Fig. 5A–G). Of note was the large decrease in mean SJC66 scores from baseline to month 6 (5.8 to 2.1 in patients < 60 years and 5.9 to 2.9 in patients ≥ 60 years (Supplementary Fig. 5B)) and the large decrease in mean PGA-PsA scores from baseline to month 6 (53.8 to 29.7 in patients < 60 years and 53.4 to 29.6 in patients ≥ 60 years (Supplementary Fig. 5E)).

### Treatment persistence

The proportion of patients who remained on ustekinumab treatment to 36 months was numerically lower in patients < 60 years, with more patients 173/336 (51.5%; 95% CI 46.0, 56.9) < 60 years stopping or switching vs 47/103 (45.6%; 95% CI 35.8, 55.7) patients ≥ 60 years, although the 95% CIs showed large overlap, with a mean (SD) treatment duration of 24.22 (12.39) months vs 26.11 (11.82) months, respectively.

**Fig. 4:**
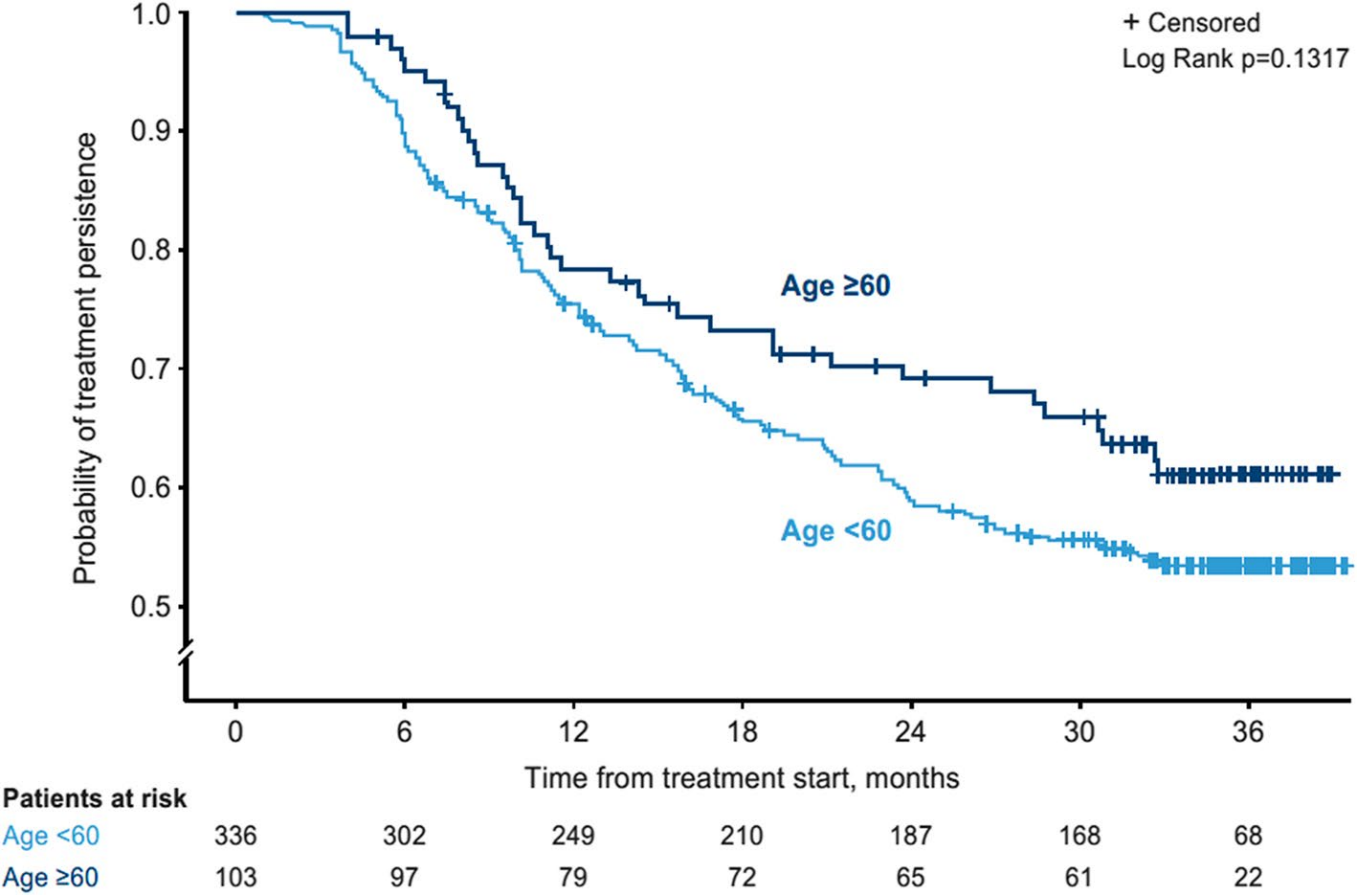
Kaplan-Meier plot of treatment persistence (time to ustekinumab treatment stop/switch) over time and by age subgroup

The Kaplan-Meier time to initial treatment stop/switch was similar between age groups (mean of 1.98 [95% CI 1.88, 2.09] vs 2.16 [95% CI 1.99, 2.33] years, for patients < 60 years vs ≥ 60 years, respectively) (Fig. 4).

The most common reasons for ceasing ustekinumab treatment were lack of effectiveness: patients < 60 years 121/142 (85.2%; 95% CI 78.3, 90.6) vs ≥ 60 years 22/30 patients (73.3%; 95% CI 54.1, 87.7) and safety/tolerability concerns: < 60 years 22/142 patients (15.5%; 95% CI 10.0, 22.5) vs ≥ 60 years 7/30 (23.3%; 95% CI 9.9, 42.3).

## Discussion

There is a need for a well-tolerated and effective bDMARD therapy for PsA that can be confidently utilised for older patients with multiple potential comorbidities. In patients taking ustekinumab, fewer AEs were observed over 3 years for younger versus older patients with PsA and ustekinumab was effective in both age groups (< 60 years vs ≥ 60 years). Persistence was numerically higher in the older age group.

Older patients are more likely to have comorbidities, such as CV disease, compared with younger patients [12]. Previous studies in older patients with rheumatoid arthritis or PsA have shown that they have an increased probability of suffering a first AE earlier after starting biologic treatment compared with younger patients [2]. Real-world evidence of the safety and effectiveness of treatments in older subgroups is therefore valuable for guiding treatment decisions and managing care in this population of patients who are more likely to suffer from age-related disorders.

Over the course of this three-year study, an acceptable safety profile in both age groups was maintained. The majority of AEs of special interest were infections (serious or opportunistic [with possible relatedness]) in patients < 60 years and CV events in patients ≥ 60 years. For the older group this is as expected, given the high level (79%) of existing CV/metabolic disorders at baseline.

PsA can affect a patient’s physical and mental well-being [13, 14]. This analysis indicated improvements at 6 months in cDAPSA, MDA, dactylitis, and skin and nail involvement, which were generally maintained over the three years. Disease activity (as measured by MDA) and disease impact (as measured by PsAID) scores suggest that patients ≥ 60 years may reach a plateau from approximately month 6 onwards (as opposed to younger patients who appear to continue to improve over time). This likely reflects that patients ≥ 60 years are affected by many other factors and diseases outside of their PsA condition, that may interact with their disease activity and impact [15]. Overall, the improvements seen in other patient-reported outcomes (including HAQ-DI, PtGA-VAS and patient assessment of pain) suggest that both age subgroups experience treatment benefits with respect to signs and symptoms of disease. This long-term analysis by age subgroup should allow for more confidence in the use of ustekinumab in older patients.

Other studies have examined the persistence of biologic treatments in PsA patients. For example, in a population-based study in Sweden, ustekinumab exhibited a favourable treatment persistency profile relative to adalimumab [16]. In the analysis presented here, ustekinumab tended to show better persistence in patients ≥ 60 years of age compared with patients < 60 years of age. Safety is often the primary consideration influencing the choice of bDMARD given to an older patient group by healthcare professionals and concerns about co-morbidities may bias selection towards ustekinumab as this is generally well tolerated. Similarly, older patients themselves may be inclined to continue with a drug they tolerate well rather than switching to another treatment with unknown tolerability. In addition, ustekinumab appears to have a better safety profile than other biologics as demonstrated in an analysis of the 2014 Psoriasis Longitudinal Assessment and Registry (PSOLAR) data [17].

The real-world nature of PsABio has the advantage of providing data from a less highly selected, homogeneous patient population than randomised controlled trials [18]. However, as with all real-world studies, limitations include inconsistencies in collecting electronic data and missing data. A limitation specific to this post-hoc analysis, is that when separating by subgroups, the number of patients were considerably diminished for some outcomes, leaving a small sample size for patients ≥ 60 years such that the resultant confidence intervals were wide and overlapping for many data points. Another limitation is that adverse events were recorded via patient recall at each 6-month visit so may not be a completely accurate record.

## Conclusions

In this real-world study, no clinically meaningful treatment-related differences were observed in safety, effectiveness or treatment persistence of ustekinumab over 3 years between younger (< 60 years) and older (≥ 60 years) subgroups of patients with PsA. Although fewer AEs were observed over 3 years for younger versus older patients with PsA, persistence was numerically higher in the older age group. These data provide reassurance regarding the use of ustekinumab in an older, potentially more vulnerable, patient population.

## Supplementary Information

**Additional file 1: Supplementary Figure 1.** Incidence rates per 100 patient-years of adverse events of special interest in patients receiving ustekinumab. **Supplementary Figure 2.** Proportion of patients achieving resolution of dactylitis^≠^, over time and according to age subgroup, % (95% CI). **Supplementary Figure 3.** Proportion of patients achieving resolution of nail lesions^≠^, over time and according to age subgroup, % (95% CI). **Supplementary Figure 4.** Proportion of patients with skin involvement: body surface area, over time and according to age subgroup, %. **Supplementary Figure 5A.** Change in total tender joint count, 68 joints, over time and according to age subgroup, Mean (95% CI). **Supplementary Figure 5B.** Change in total swollen joint count, 66 joints, over time and according to age subgroup, Mean (95% CI). **Supplementary Figure 5C.** Change in health assessment questionnaire – disability index, over time and according to age subgroup, Mean (95% CI). **Supplementary Figure 5D.** Change in C-reactive protein concentration, over time and according to age subgroup, Mean, mg/dL (95% CI). **Supplementary Figure 5E.** Change in physician’s global assessment of disease activity, over time and according to age subgroup, Mean, mm (95% CI). **Supplementary Figure 5F.** Change in patient’s global assessment of disease activity-VAS, over time and according to age subgroup, Mean, mm (95% CI). Supplementary Figure 5G. Change in patient’s assessment of pain-VAS, over time and according to age subgroup, Mean, mm (95% CI).

## Data Availability

No data are available. Access to anonymised individual participant-level data will not be provided for this trial as it meets one or more of the exceptions described on https://yoda.yale.edu/ under “Data Use Agreement—Janssen Pharmaceuticals DUA”.

## Abbreviations

ACR: American College of Rheumatology
AE: Adverse event
bMARDs: Biologic disease-modifying antirheumatic drugs
cDAPSA: Clinical Disease Activity Index for Psoriatic Arthritis
CI: Confidence interval
CRP: C-reactive protein
CV: Cardiovascular
HAQ-DI: Health Assessment Questionnaire-Disability Index
LDA: Low disease activity
LOCF: Last observation carried forward
MDA: Minimal disease activity
Pain-VAS: Patient Assessment of Pain VAS
PGA-PsA: Physician’s Global Assessment of Disease Activity
PsA: Psoriatic arthritis
PsAID-12: Psoriatic Arthritis Impact of Disease-12
PtGA-VAS: Patient’s Global Assessment of Disease Activity-Visual Analogue Scale
SAE: Serious adverse event
SJC: Swollen joint count
TJC: Tender joint count
TNFi: Tumour necrosis factor inhibitors
VLDA: Very low disease activity

## References

[1] National Psoriasis Foundation. About Psoriasis. 2021. https://www.psoriasis.org/. Accessed 1 May 2022.

[2] Vela P, Sanchez-Piedra C, Perez-Garcia C, Castro-Villegas MC, Freire M, Mateo L (2020). Influence of age on the occurrence of adverse events in rheumatic patients at the onset of biological treatment: data from the BIOBADASER III register. Arthritis Res Ther.

[3] Genc Yavuz B, Colak S, Guven R, Altundag İ, Seyhan AU, Gunay IR (2021). Clinical features of the 60 years and older patients infected with 2019 novel coronavirus: can we predict mortality earlier?. Gerontology.

[4] Kimball AB, Papp KA, Wasfi Y, Chan D, Bissonnette R, Sofen H (2013). Long-term efficacy of ustekinumab in patients with moderate-to-severe psoriasis treated for up to 5 years in the PHOENIX 1 study. J Eur Acad Dermatol Venereol.

[5] Langley RG, Lebwohl M, Krueger GG, Szapary PO, Wasfi Y, Chan D (2015). Long-term efficacy and safety of ustekinumab, with and without dosing adjustment, in patients with moderate-to-severe psoriasis: results from the PHOENIX 2 study through 5 years of follow-up. Br J Dermatol.

[6] Kavanaugh A, Ritchlin C, Rahman P, Puig L, Gottlieb AB, Li S (2014). Ustekinumab, an anti-IL-12/23 p40 monoclonal antibody, inhibits radiographic progression in patients with active psoriatic arthritis: results of an integrated analysis of radiographic data from the phase 3, multicentre, randomised, double-blind, placebo-controlled PSUMMIT-1 and PSUMMIT-2 trials. Ann Rheum Dis.

[7] Ritchlin C, Rahman P, Kavanaugh A, McInnes IB, Puig L, Li S (2014). Efficacy and safety of the anti-IL-12/23 p40 monoclonal antibody, ustekinumab, in patients with active psoriatic arthritis despite conventional non-biological and biological anti-tumour necrosis factor therapy: 6-month and 1-year results of the phase 3, multicentre, double-blind, placebo-controlled, randomised PSUMMIT 2 trial. Ann Rheum Dis.

[8] Smolen JS, Siebert S, Korotaeva TV, Selmi C, Bergmans P, Gremese E (2021). Effectiveness of IL-12/23 inhibition (ustekinumab) versus tumour necrosis factor inhibition in psoriatic arthritis: observational PsABio study results. Ann Rheum Dis.

[9] Gossec L, Siebert S, Bergmans P, de Vlam K, Gremese E, Joven-Ibáñez B (2022). Persistence and effectiveness of the IL-12/23 pathway inhibitor ustekinumab or tumour necrosis factor inhibitor treatment in patients with psoriatic arthritis: 1-year results from the real-world PsABio study. Ann Rheum Dis.

[10] Gossec L, Siebert S, Bergmans P, de Vlam K, Gremese E, Joven-Ibáñez B et al. Long-term effectiveness and persistence of ustekinumab and TNF inhibitors in patients with psoriatic arthritis: final 3-year results from the PsABio real-world study. Ann Rheum Dis. 2023;82(4):496–506. 10.1136/annrheumdis-2022-222879. 10.1136/ard-2022-222879 PMC10086293 36600178

[11] Gossec L, de Wit M, Kiltz U, Braun J, Kalyoncu U, Scrivo R (2014). A patient-derived and patient-reported outcome measure for assessing psoriatic arthritis: elaboration and preliminary validation of the Psoriatic Arthritis Impact of Disease (PsAID) questionnaire, a 13-country EULAR initiative. Ann Rheum Dis.

[12] Fragoulis GE, Nikiphorou E, McInnes IB, Siebert S (2022). Does Age Matter in Psoriatic Arthritis? A Narrative Review. J Rheumatol.

[13] Gudu T, Gossec L (2018). Quality of life in psoriatic arthritis. Expert Rev Clin Immunol.

[14] Gossec L, Walsh JA, Michaud K, Holdsworth E, Peterson S, Meakin S (2022). Effect of Fatigue on Health-Related Quality of Life and Work Productivity in Psoriatic Arthritis: Findings From a Real-World Survey. J Rheumatol.

[15] Lucasson F, Balanescu A, Kiltz U, Leung YY, Aydin SZ, Gaydukova I et al. Residual patient-reported burden in 444 patients with psoriatic arthritis in remission or low disease: A cross-sectional analysis. Joint Bone Spine. 2022;89(5):105372. 10.1016/j.jbspin.2022.105372. 10.1016/j.jbspin.2022.105372 35257864

[16] Geale K, Lindberg I, Paulsson EC, Wennerström ECM, Tjärnlund A, Noel W et al. Persistence of biologic treatments in psoriatic arthritis: a population-based study in Sweden. Rheumatol Adv Pract. 2020;4(2):rkaa070. 10.1093/rap/rkaa070. 10.1093/rap/rkaa070 PMC7772250 33409449

[17] Papp K, Gottlieb AB, Naldi L, Pariser D, Ho V, Goyal K (2015). Safety surveillance for ustekinumab and other psoriasis treatments from the Psoriasis Longitudinal Assessment and Registry (PSOLAR). J Drugs Dermatol.

[18] Vandendorpe AS, de Vlam K, Lories R. Evolution of psoriatic arthritis study patient population characteristics in the era of biological treatments. RMD Open. 2019;5(1):e000779. 10.1136/rmdopen-2018-000779. 10.1136/rmdopen-2018-000779 PMC6347028 30740243

