# Supplementary material for: Response to treatment in psoriatic arthritis, the effect of age: analysis of patients receiving ustekinumab in the PsABio real-world study
Source: Arthritis Res Ther. 2023 Jun 9;25:100. doi: 10.1186/s13075-023-03078-8 (PMC10251537; doi:10.1186/s13075-023-03078-8)
Supplement: Supplementary file 1 — Additional file 1: Supplementary Figure 1. Incidence rates per 100 patient-years of adverse events of special interest in patients receiving ustekinumab. Supplementary Figure 2. Proportion of patients achieving resolution of dactylitis≠, over time and according to age subgroup, % (95% CI). Supplementary Figure 3. Proportion of patients achieving resolution of nail lesions≠, over time and according to age subgroup, % (95% CI). Supplementary Figure 4. Proportion of patients with skin involvement: body surface area, over time and according to age subgroup, %. Supplementary Figure 5A. Change in total tender joint count, 68 joints, over time and according to age subgroup, Mean (95% CI). Supplementary Figure 5B. Change in total swollen joint count, 66 joints, over time and according to age subgroup, Mean (95% CI). Supplementary Figure 5C. Change in health assessment questionnaire – disability index, over time and according to age subgroup, Mean (95% CI). Supplementary Figure 5D. Change in C-reactive protein concentration, over time and according to age subgroup, Mean, mg/dL (95% CI). Supplementary Figure 5E. Change in physician’s global assessment of disease activity, over time and according to age subgroup, Mean, mm (95% CI). Supplementary Figure 5F. Change in patient’s global assessment of disease activity-VAS, over time and according to age subgroup, Mean, mm (95% CI). Supplementary Figure 5G. Change in patient’s assessment of pain-VAS, over time and according to age subgroup, Mean, mm (95% CI). [file 13075_2023_3078_MOESM1_ESM.docx]

**Supplementary material**

**Supplementary Figure 1. Incidence rates per 100 patient-years of adverse events of special interest in patients receiving ustekinumab**

**
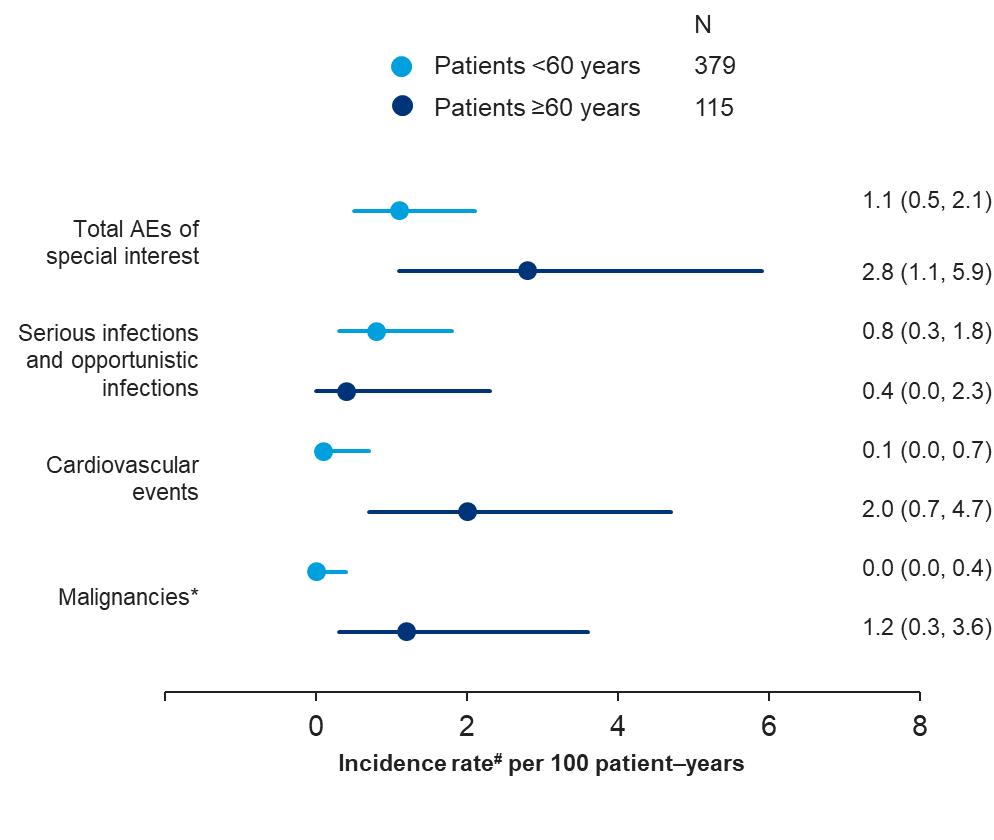
**

AE, adverse event. AEs do not include neoplasms.

*Malignancies incidence rate was analysed using 12-month lag time; ^#^Exposure-adjusted incidence rate calculated as the number of events divided by the sum of the individual exposure times.

**Supplementary Figure 2. Proportion of patients achieving resolution of dactylitis^≠^, over time and according to age subgroup, % (95% CI)**


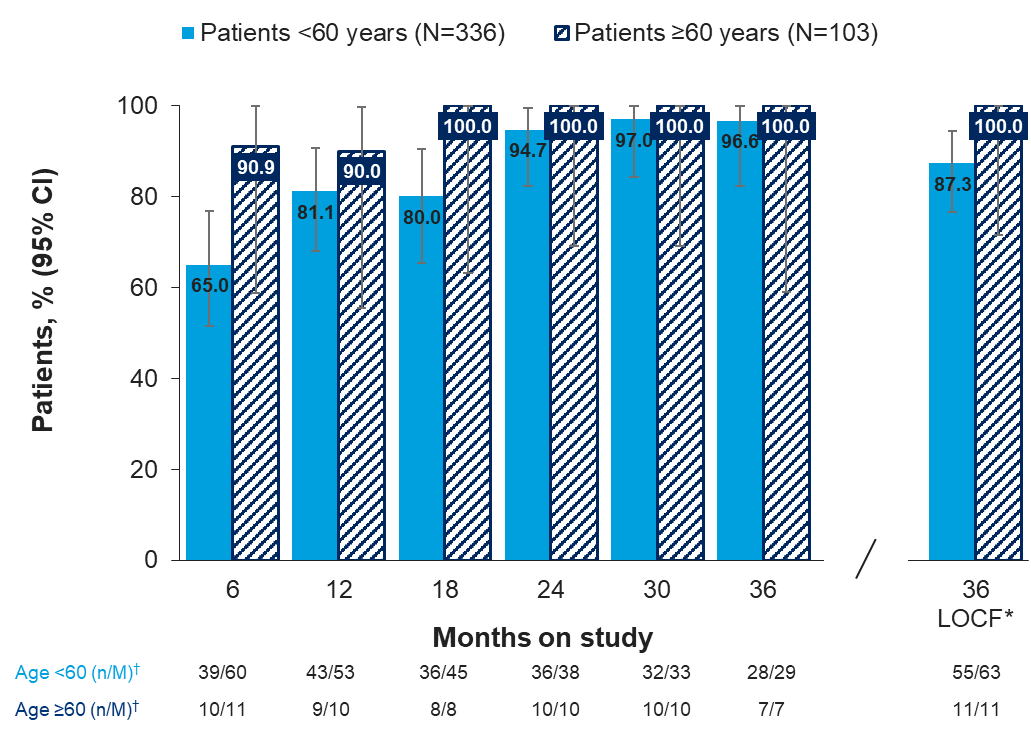


CI, confidence interval; LOCF, last observation carried forward.

**^≠^**63/315 (20.0%) patients <60 years and 11/93 (11.8%) patients ≥60 years had dactylitis at baseline.

*last observation carried forward, all other bars show observed case analysis; ^†^n, number of patients achieving resolution of dactylitis; M, number of patients with an assessment at that specific time point.

**Supplementary Figure 3. Proportion of patients achieving resolution of nail lesions^≠^, over time and according to age subgroup, % (95% CI)**


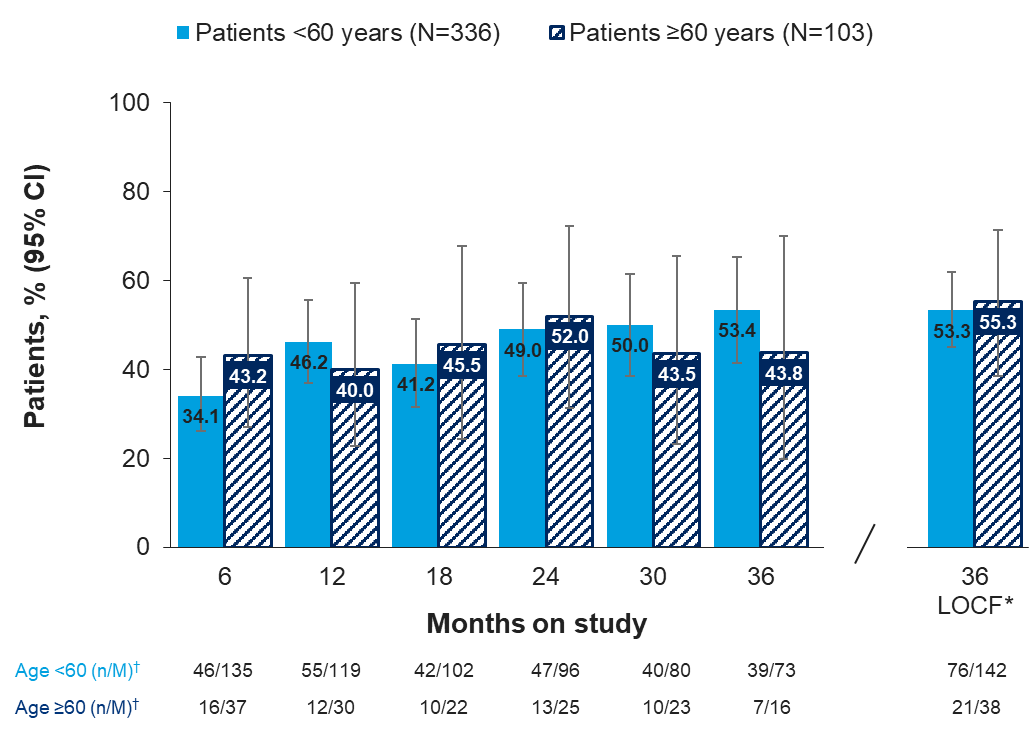


CI, confidence interval; LOCF, last observation carried forward.

^≠^142/304 (46.7%) patients <60 years and 38/88 (43.2%) patients ≥60 years had nail lesions at baseline.

*last observation carried forward, all other bars show observed case analysis; ^†^n, number of patients achieving resolution of nail lesions; M, number of patients with an assessment at that specific time point.

**Supplementary Figure 4. Proportion of patients with skin involvement: body surface area, over time and according to age subgroup, %**


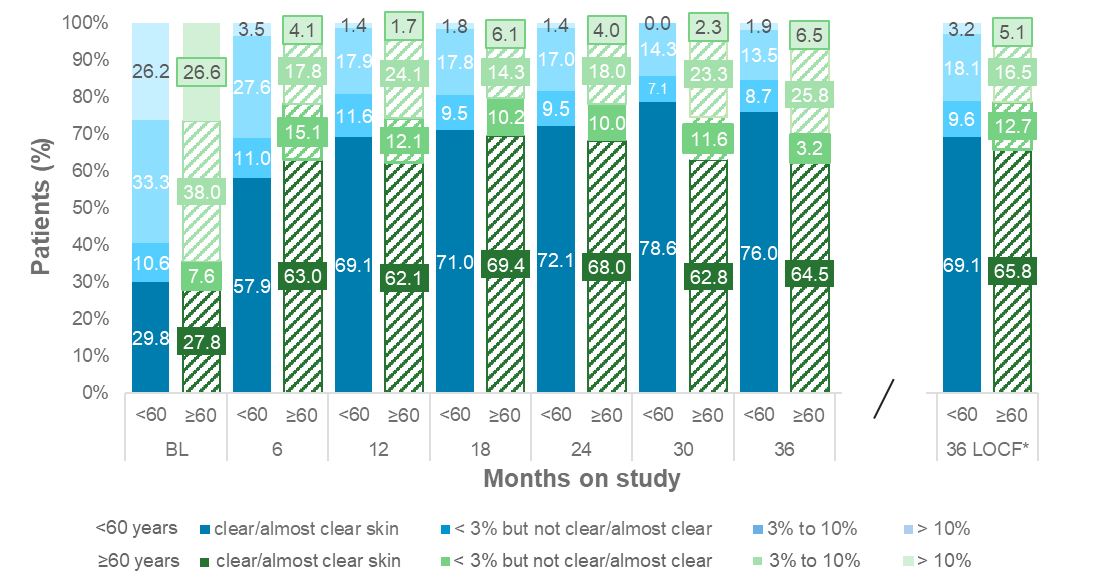


BL, baseline; LOCF, last observation carried forward; <60, patients <60 years; ≥60, patients ≥60 years.

*last observation carried forward; all other bars show observed case analysis.

**Supplementary Figure 5A. Change in total tender joint count, 68 joints, over time and according to age subgroup, Mean (95% CI)**

**
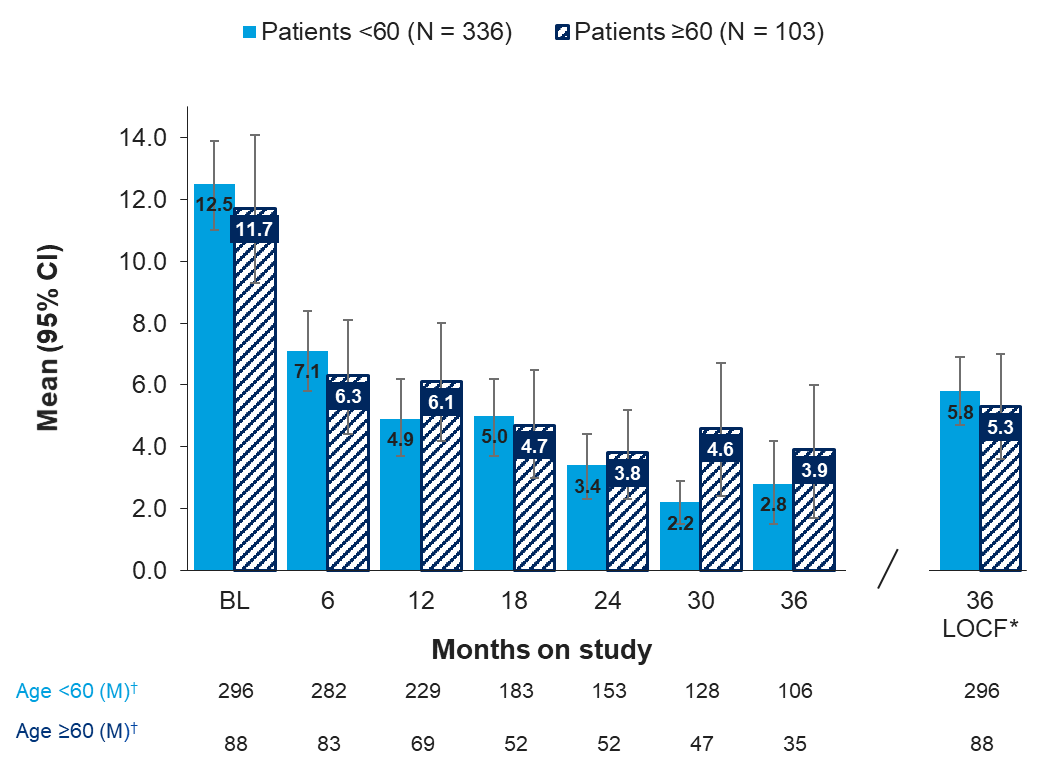
**

BL, baseline; CI, confidence interval; LOCF, last observation carried forward.

*last observation carried forward, all other bars show observed case analysis; ^†^M, number of patients with an assessment at that specific time point.

**Supplementary Figure 5B. Change in total swollen joint count, 66 joints, over time and according to age subgroup, Mean (95% CI)**

**
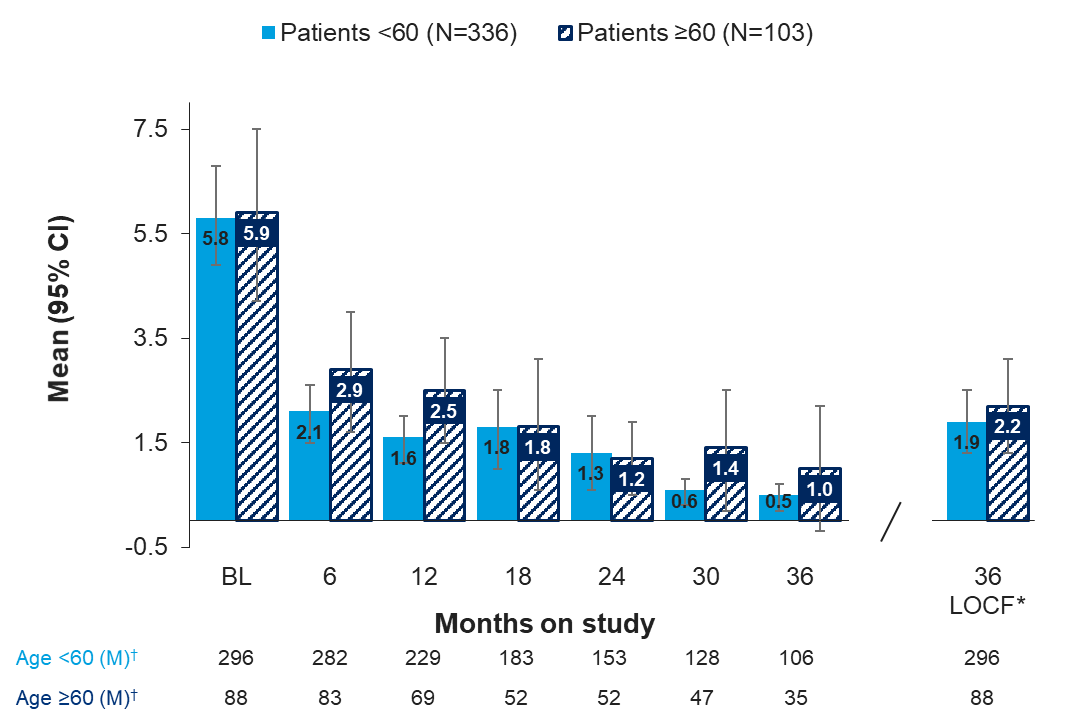
**

BL, baseline; CI, confidence interval; LOCF, last observation carried forward.

*last observation carried forward, all other bars show observed case analysis; ^†^M, number of patients with an assessment at that specific time point.

**Supplementary Figure 5C. Change in health assessment questionnaire – disability index, over time and according to age subgroup, Mean (95% CI)**


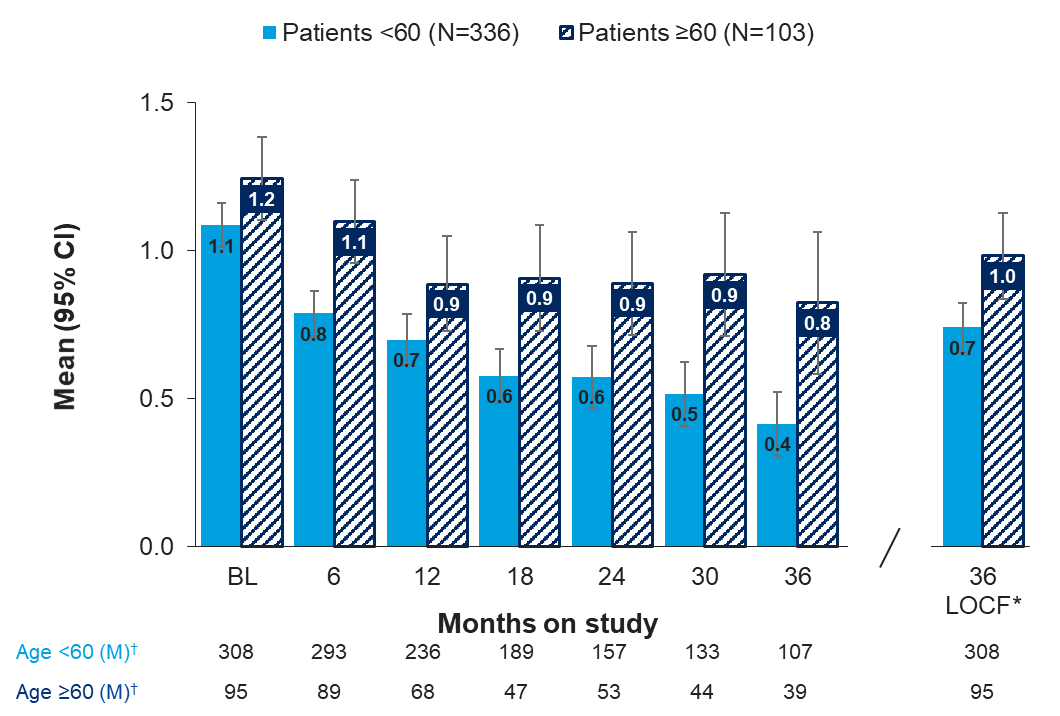


BL, baseline; CI, confidence interval; LOCF, last observation carried forward.

*last observation carried forward, all other bars show observed case analysis; ^†^M, number of patients with an assessment at that specific time point.

**Supplementary Figure 5D. Change in C-reactive protein concentration, over time and according to age subgroup, Mean, mg/dL (95% CI)**


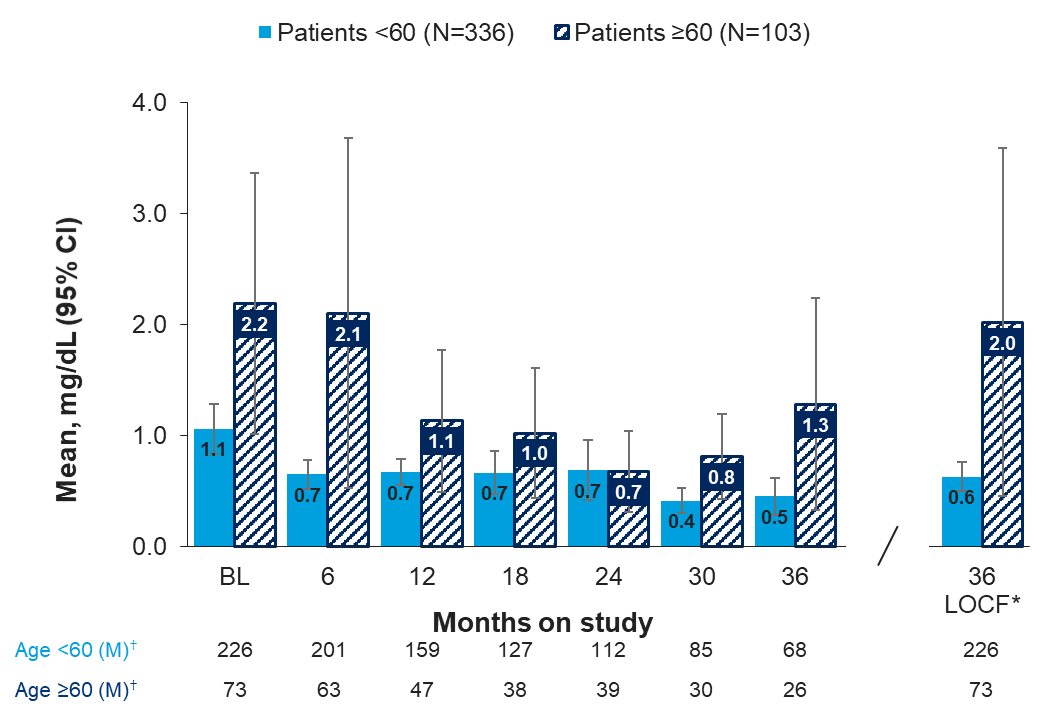


BL, baseline; CI, confidence interval; LOCF, last observation carried forward.

*last observation carried forward, all other bars show observed case analysis; ^†^M, number of patients with an assessment at that specific time point.

**Supplementary Figure 5E. Change in physician’s global assessment of disease activity, over time and according to age subgroup, Mean, mm (95% CI)**


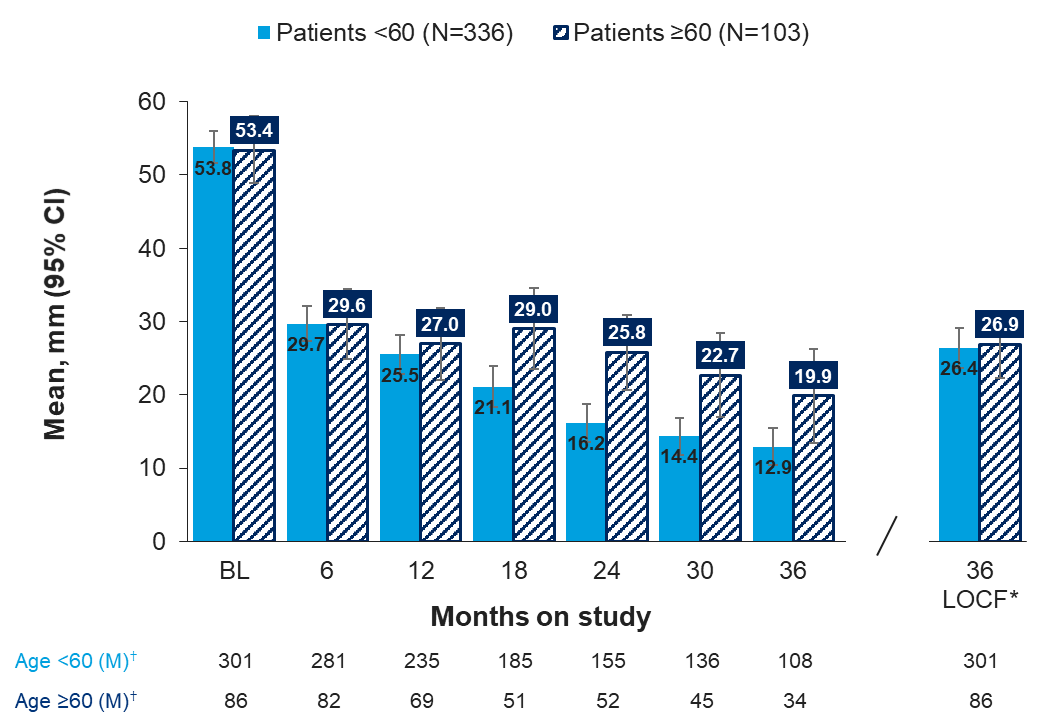


BL, baseline; CI, confidence interval; LOCF, last observation carried forward.

*last observation carried forward, all other bars show observed case analysis; ^†^M, number of patients with an assessment at that specific time point.

**Supplementary Figure 5F. Change in patient’s global assessment of disease activity-VAS, over time and according to age subgroup, Mean, mm (95% CI)**


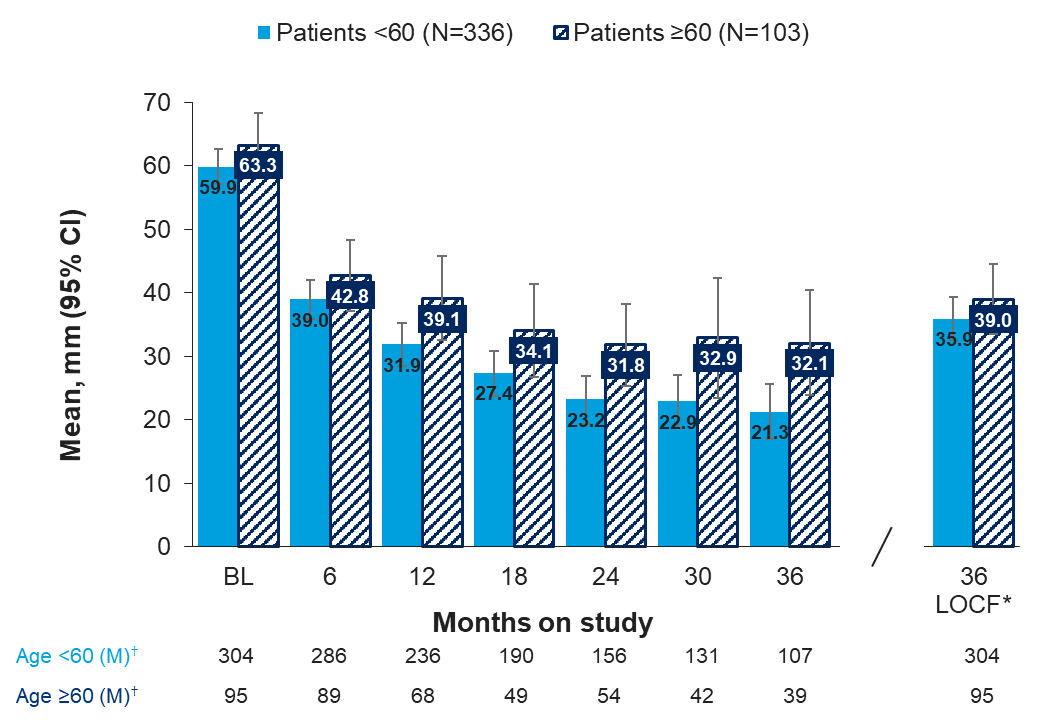


BL, baseline; CI, confidence interval; LOCF, last observation carried forward; VAS, visual analogue scale.

*last observation carried forward, all other bars show observed case analysis; ^†^M, number of patients with an assessment at that specific time point.

**Supplementary Figure 5G. Change in patient’s assessment of pain-VAS, over time and according to age subgroup, Mean, mm (95% CI)**


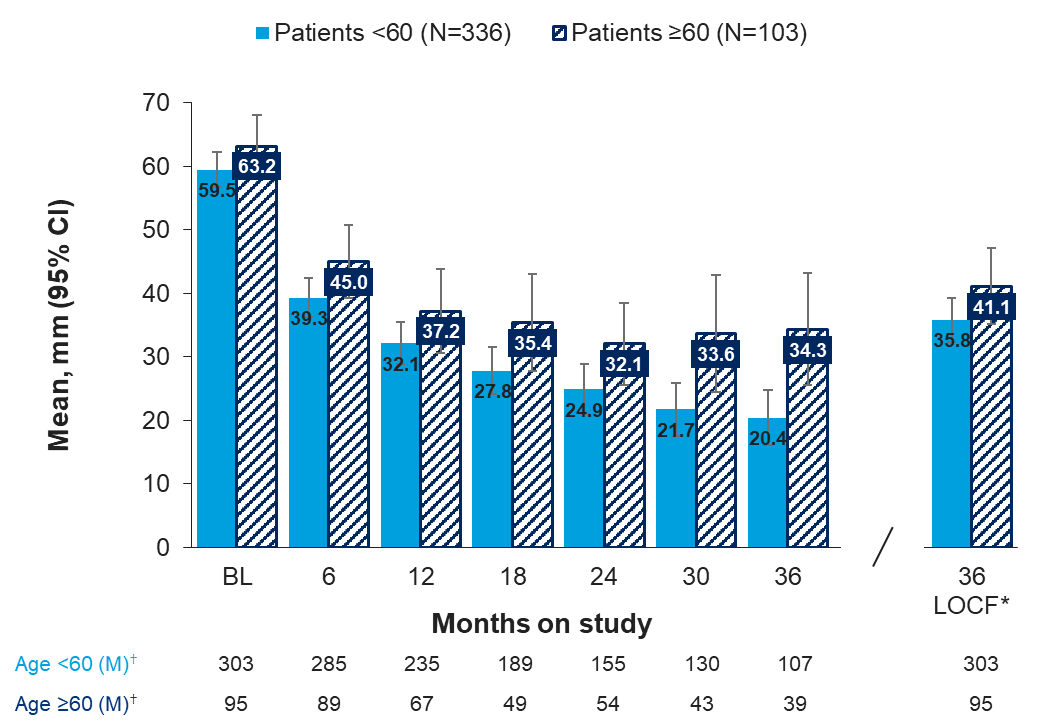


BL, baseline; CI, confidence interval; LOCF, last observation carried forward; VAS, visual analogue scale.

*last observation carried forward, all other bars show observed case analysis; ^†^M, number of patients with an assessment at that specific time point.
